# Structuring data analysis projects in the Open Science era with Kerblam!

**DOI:** 10.12688/f1000research.157325.2

**Published:** 2025-04-04

**Authors:** Luca Visentin, Luca Munaron, Federico Alessandro Ruffinatti

**Affiliations:** 1 Department of Life Sciences and Systems Biology, University of Turin, Turin, 10136, Italy

**Keywords:** Project Management, reproducibility, open science, workflows, data management

## Abstract

**Background:**

Structuring data analysis projects, that is, defining the layout of files and folders needed to analyze data using existing tools and novel code, largely follows personal preferences. Open Science calls for more accessible, transparent and understandable research. We believe that Open Science principles can be applied to the way data analysis projects are structured.

**Methods:**

We examine the structure of several data analysis project templates by analyzing project template repositories present in GitHub. Through visualization of the resulting consensus structure, we draw observations regarding how the ecosystem of project structures is shaped, and what salient characteristics it has.

**Results:**

Project templates show little overlap, but many distinct practices can be highlighted. We take them into account with the wider Open Science philosophy to draw a few fundamental Design Principles to guide researchers when designing a project space. We present Kerblam!, a project management tool that can work with such a project structure to expedite data handling, execute workflow managers, and share the resulting workflow and analysis outputs with others.

**Conclusions:**

We hope that, by following these principles and using Kerblam!, the landscape of data analysis projects can become more transparent, understandable, and ultimately useful to the wider community.

## Introduction

Data analysis is a key step in all scientific experiments. In numerical data-centric fields, it is, in essence, a series of computational steps in which input data is processed by software to produce some output. Usually, the ultimate goal is to create secondary data for human interpretation to produce knowledge and insight into some phenomena. These manipulations can involve downloading input data on local storage, creating workflows and novel software—also saved locally—and running the analysis on local or remote (“cloud”) hardware.

The reproducibility of scientific output is a hot topic in recent years. A scoping report by the European Commission ^1^ covered this issue in 2020, highlighting results from a popular survey by Nature in 2016, ^2^ where researchers reported different success rates when trying reproducing experiments, with lowest scores in areas such as Chemistry, Biology and Medicine. Such failure rates may be due to multiple “failure points”: the complexity of the experimental design being so high as to be irreplicable, missing protocols and other procedures, unavailable input data or analysis code, bad computational reproducibility ascribable to, for example, versioning of packages, etcetera. We believe that one such failure point relates to the structure of the data analysis projects, and the way they are packaged and shown to the public.

In this article, we will use the phrase “data analysis project structure” to refer to the way data analysis projects are organized on the actual file system, including the structure of folders on disk, the places where data, code, and workflows are stored, and the format in which the project is shared with the public. Unfortunately, as we will later demonstrate, such structures can vary considerably among researchers, making it difficult for the public to inspect and understand them.

With the Open Science movement gaining traction in recent years, ^3^ there is a growing need to standardize how routine data analysis is structured and carried out. A significant milestone in the application of Open Science philosophy to practice are the FAIR principles, ^4^ which propose characteristics that data should have to be more useful to the wider public. Notably, even though originally thought to provide guidelines for the management of data, FAIR principles have recently been extended to other contexts, such as software. ^5^ By making data analyses more transparent and intelligible, the standardization of project structure complies with the FAIR principles’ call for more Findable, Accessible, Interoperable, and Reusable research objects. ^4^ Efforts are being made from many parts to make reproducible pipelines easier to create and execute by the wider public—for example, by leveraging methods such as containerization. ^6– 8^ However, while new tools and technologies offer unprecedented opportunities to make the whole process of data analysis increasingly transparent and reproducible, their usage still requires time and effort, as well as expertise and sensibility to the issue of standardization and reproducibility by the researcher.

In this work, we inspect the structure of many data science and data analysis project templates that are currently available online. Then, we outline best practices and considerations to take into account when thinking about structuring data analysis projects. Following these principles, we propose a simple, lightweight, and extensible project structure that fits many needs and is in line with projects already present in the ecosystem, thus providing a certain level of standardization. Finally, we introduce Kerblam!, a new tool that can be used to work in projects with this standard structure, taking care of common tasks, such as data retrieval and cleanup, workflow management, and containerization support. This could ultimately benefit the scientific community by making others’ work easier to understand and reproduce, for example, during the peer-review process. We hope that this article will be useful to both established data analysts, prompting them to streamline their data analysis projects, and researchers willing to increase the reproducibility of their data analysis efforts.

## Data collection

To fetch the structure of the most common data analysis projects, we ran two GitHub searches: one for the keywords *cookiecutter* and *data* ( cookiecutter is a Python package that allows users to create, or “cut,” new projects from templates) and the other for the much more generic keywords *project* and *template.*

We downloaded the top 50 repositories from each search sorted by GitHub stars as proxies for popularity and adoption rate. For each project, we either cut it with the cookiecutter Python package or used it as is (for non-cookiecutter templates). Of these 100 repositories, 87 could ultimately be successfully cut and parsed and were therefore considered. All files and folders from the resulting projects were listed and compiled into a frequency graph.

Some housekeeping files (like the .git directory and all its content) were stripped from the final search results, as they were deemed irrelevant to the project as a whole. For example, .gitkeep files, which are commonly used to commit empty directories to version control, were excluded from the final figure. Finally, only files present in at least three or more templates were retained for plotting.

The analysis was performed with the latest commits of all considered repositories as of the 12th of July 2024. The only exception was the “drivendataorg/cookiecutter-data-science” repository, for which we fetched version 1.0 due to the non-standard parsing requirements of the latest commit.

The code for this analysis is available online. See the “Software availability statement” section for more information.

## Data interpretation

**Figure 1.**
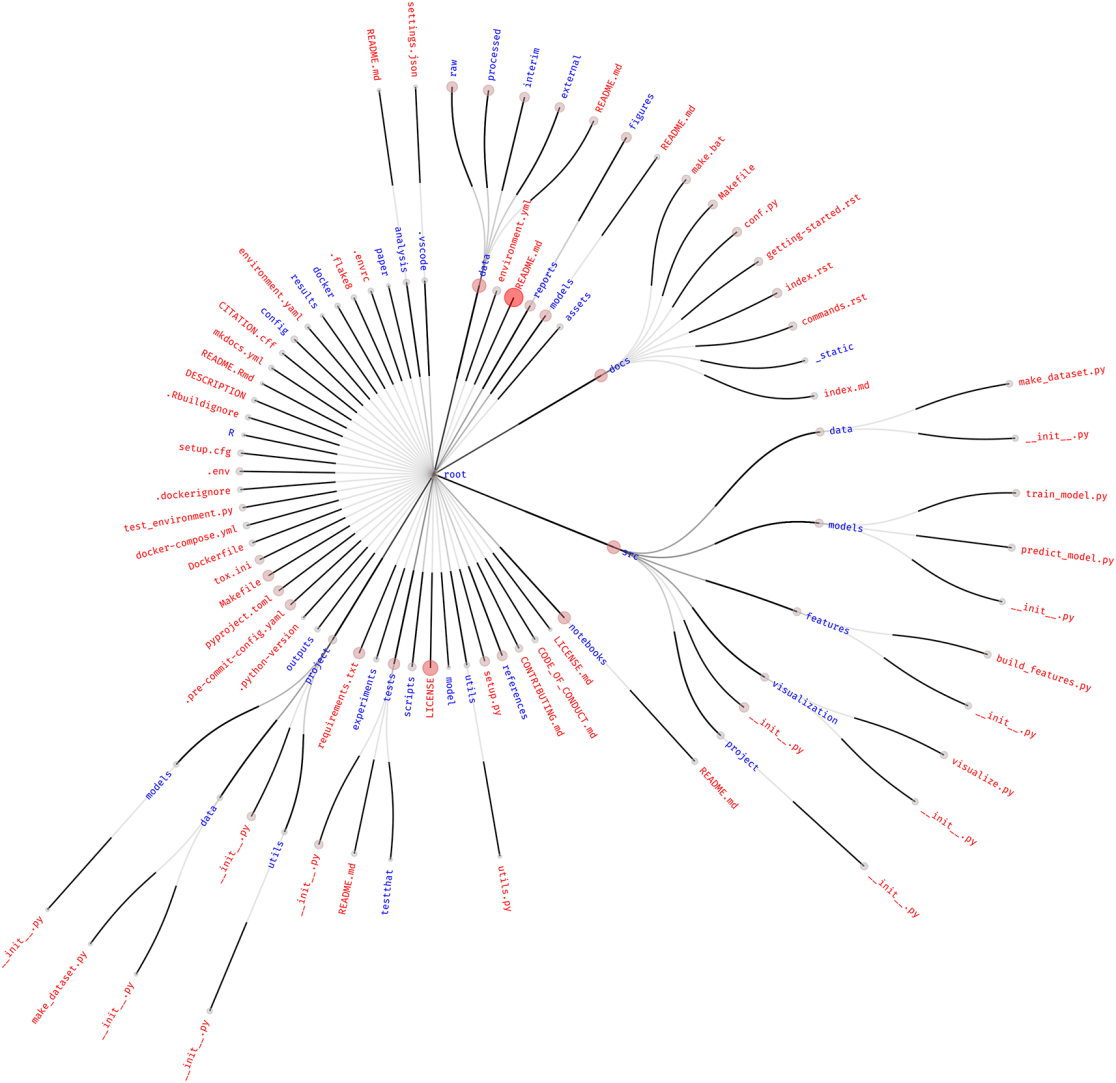
Frequency graph of the structure of the 87 most starred data analysis project templates. Only files present in at least three or more templates are shown, as retrieved from GitHub. The size and color intensity of the circle at the tip of each link is proportional to the frequency with which that file or folder is found in different project templates. Red text represents files, while blue text represents folders. The central dot of the root node was assigned an arbitrary size.

The choice of how to structure projects is an issue universally shared by anyone who performs data analysis. This results in a plethora of different tools, folder hierarchies, accepted practices, and customs. To explore the most common practices, we inspected 87 different project templates available on GitHub and produced a frequency graph of shared files and folders, as shown in Figure 1.

By looking at this figure, we can point out common patterns in project structuring. However, it must be noted that templates influence each other. For example, many Python data science project templates seem to be modified versions of drivendataorg/cookiecutter-data-science, which has a very high number of stars and is, therefore, probably popular with the community.

In any case, the two most highly found files are the README.md file (with a frequency of $\frac{77}{87}≃0.89$ ) and the LICENSE and LICENSE.md files ( $\frac{46+3}{87}≃0.56$ ).

The pyproject.toml file at the top level of the repository, which marks the project as a Python package, is also prevalent ( $\frac{16}{87}≃0.18$ ). This is potentially due to the popular “cookiecutter-data-science” template mentioned before, also highlighting how projects following this template are intimately linked with the usage of Python, potentially exclusively. The predominance of Python-based projects is also noticeable by the presence of requirements.txt (a file usually used to store Python’s package dependencies), setup.py, and setup.cfg (now obsolete versions of the pyproject.toml file used to configure Python’s build system). The project folder at the top level of the templates is most likely the Python package (represented by the __init__.py file) that the pyproject.toml file refers to (the name “project” is artificial, deriving from the default way that cookiecutter templates were cut).

The presence of files related to the R programming language (the R directory, .Rbuildignore, README.Rmd) reflects its usage in the data analysis field, although at a lower frequency than Python. The relatively low prevalence of the R programming language could be due to biases introduced by the search queries or to the overwhelming popularity of Python project templates, as well as the fact that the cookiecutter utility itself is written in Python.

Community-relevant files such as CONTRIBUTING.md ( $\frac{8}{87}≃0.09$ ) and CODE_OF_CONDUCT.md ( $\frac{5}{87}≃0.06$ ) show little prevalence in templates. This is also true for the CITATION.cff ( $\frac{4}{87}≃0.05$ ) file, which is useful for machine-readable citation data.

The src ( $\frac{31}{87}≃0.36$ ), data ( $\frac{35}{87}≃0.40$ ), and docs ( $\frac{28}{87}≃0.32$ ) folders are highly represented, containing code, data, and project documentation, respectively. In particular, the data directory contains with a high frequency the raw, processed, interim, and external folders to host the different data types—input, output, intermediate, and third party—according to the structure promoted by the “cookiecutter-data-science” template. The prevalence of these sub-folders, however, is lower than the frequency of data itself, which means that the presence of the data folder is not uniquely due to that specific template. Interestingly, other templates include data in the src folder, mixing it with the analysis code. Other common folders present in the src directory are also the ones promoted by “cookiecutter-data-science,” but again, as already noted for data, their occurrence is lower than that of the parent folder, indicating that many different templates adopt src as a folder name.

Docker-related files are present, mostly at the top level of the project: Dockerfile ( $\frac{5}{87}≃0.06$ ), .dockerignore ( $\frac{4}{87}≃0.05$ ), and docker-compose.yml or yaml ( $\frac{6+1}{87}≃0.08$ ). Docker-related files and folders are also present with sub-threshold frequencies in many other forms, often as directories with multiple Dockerfiles in different folders. The presence of the docker-compose.yml file and docker subdirectories could be indicative of a common need to manage multiple execution environments—that work together in the case of Docker Compose—throughout the analysis.

The sparse use of many tools can be appreciated by the number of unique files and folders across all templates. Of the 4195 different files and directories considered by this approach, the vast majority (3908, or 93.16 %) were present in only one template. Looking at directories only, 783 were unique over 864 total (90.63 %). This figure might be inflated owing to the presence of some compiled libraries, files, and Git objects that are included in the analysis and not correctly removed by our filtering. However, we argue that this overwhelmingly high uniqueness would not be significantly affected by manual filtering.

The small overlap between templates reflects that project structure is, by its nature, a matter of personal preference. Nevertheless, Figure 1 confirms that the core structure of the repositories tends to be similar. This is potentially due to both the epistemic need to share one’s own work with others and the technical requirements of research tools, which cause the adoption of community standards either by choice (in the former case) or imposition (in the latter). For instance, the high presence of the README.md file is a community standard that is broadly shared by the majority of software developers, users, and researchers alike. This adoption is purely for practical reasons: specifically, the need to share the description of the work with others in an obvious (“please read me”), logical (in the topmost layer of the project layout), and predictable (i.e., used by the wider community) manner.

Borrowing a term from genetics, the README file can be thought to be a “housekeeping” file: without it, the usefulness of a project is severely impaired. In this regard, another possible housekeeping candidate is the LICENSE file. It is essential to collaborate with the community in the open-source paradigm and is thus commonly found in many software packages. The concession for code reuse is also essential in data analysis projects, both to allow reproducers to replay the initial work and for other researchers to build on previous knowledge. Incidentally, the common presence of the LICENSE file in the project *template* is interesting. This could be due to either apathy toward licensing issues, leading to picking a “default license” without many considerations, or a general feeling in individuals that one particular license fits their projects across the board.

A potentially new housekeeping file that is not yet commonly found is the CITATION.cff file. This file contains machine-readable citation metadata that can be used by both human and machine users to obtain such information, potentially automatically.

## Intervention

### Design principles

The observations made above can all be considered when designing a more broadly applicable project template that may be used in a variety of contexts. To this end, it is helpful to conceptualize some core guiding principles that should be followed by all data analysis projects, particularly under the Open Science paradigm.

Because data analysis projects often involve writing new software, a data analysis project structure requires support for both *data analysis* proper and *software development.* Software development methods fall outside the scope of this work, but some concepts are useful in the context of data analysis, particularly for *ad hoc* data analysis. For instance, many programming languages require specific folder layouts to create self-contained distributed software. For instance, to create a package with the Python programming language ( https://python.org/), a specific project layout must be followed. ^9^ This is also visible in Figure 1, with the presence of the project folder and many files specific to Python packages, crucially, in the locations required by Python build backends. Something similar occurs for many programming languages, such as R ^10^ and Rust, ^11^ among others.

However, a researcher may not want to create self-contained, distributed software. Languages such as Python and R ( https://www.r-project.org/) can interpret and execute single-file scripts to achieve certain goals (i.e., “scripting”). As scripting is fast, convenient, and easy to perform, it is the most common method of data analysis. Scripting provides flexibility during the development process; however, this typically exacerbates the fragmentation of project structures. In particular, the environment of execution now becomes much more relevant: which packages are installed and at which versions, the order in which the scripts were read and executed, and, potentially, even the order of *which lines* are (manually) run becomes important to the success of the overall analysis.

This increased flexibility is obviously useful for the research process, which requires the ability to change quickly to adapt to new findings, especially during hypothesis-generating “exploratory” research. The principles presented here aim to retain this essential requirement of adaptability but, at the same time, push for increased standardization of methods, avoiding the most common and dangerous pitfalls that can be encountered during data analysis.

*1. Use a version control system*

At its core, software is a collection of text files, and this includes data analysis software. While producing code, it is important to record the differences between the different versions of these files. This is very useful, especially during the research process, to “retrace our steps” or to attempt new methodologies without the fear of losing any previous work. Such records are also useful as provenance information and potentially as proof of authorship, similar to what a laboratory notebook does for a “wet-lab” experimental researcher.

There is consolidated software that can be used as a version control system. An overwhelming majority of projects use git ( https://git-scm.com/) for this purpose, although others exist. Platforms that integrate git, such as GitHub ( github.com) and GitLab ( gitlab.com), are increasingly used for data analysis, both as a collaboration tool during the project and as a sharing platform afterwards.

The first principle should therefore be this: **use a version control system**, such as git.

A few practical observations stem from this principle:

• version control encourages good development practices, such as atomic commits, meaningful commit messages, and more, reducing the number of mistakes made while programming, and increasing efficiency by making debugging easier;
• version control discourages the upload of very large (binary) files; therefore, input and output data cannot be efficiently shared through such a system, incentivizing the deposit of data in online archives and, by extension, favoring the FAIRness of the manipulated data objects;
• code collaboration and collaboration techniques (such as “GitHub Flow” or “trunk based development” ^12, 13^) can be useful to promote a more efficient development workflow in data analysis disciplines such as bioinformatics, especially in mid- to large-research groups;
• the core unit of a project should be a code repository, containing everything related to that project, from code to documentation, to configuration.

The use of a version control system also has implications for FAIR-ness. Leveraging remote platforms is fundamental to both Findability and Accessibility. Integrations of platforms such as GitHub with archives such as Zenodo ( https://zenodo.org/) allow developers to easily archive for long-term preservation their data analysis code, promoting Accessibility, Findability, and Reusability.

*2. Documentation is essential*

When working on a data analysis project, documentation is important for both the experimenter themselves and external users. Through ideal documentation, the rationale, process, and potentially the result of the analysis are presented to the user, together with practical steps on how to *actually* reproduce the work. As with all other aspects of data analysis, documentation takes many different forms, but is the most difficult thing to standardize for one simple reason: documentation is written by humans for human consumption. Documentation is therefore allowed high flexibility in structure, content, form, and delivery method.

Even though rigid standardization is impossible, some guidelines on how to write effective documentation can still be drawn, often from best practices in the much wider world of open-source software. We have already highlighted the fundamental role of the README file and its widespread adoption. This file contains high-level information about the project and is usually the first, and perhaps only, documentation that all users encounter and read. It is therefore essential that core aspects of the project are delivered through the README file, such as the following:

• the aim of the project, in clear, accessible language;
• methods used to achieve such an aim (and/or a link to further reading material);
• a guide on how to run the analysis on the user’s machine, potentially including information on hardware requirements, software requirements, container deployment methods, and every piece of information a human reproducer might need to execute the analysis;
• in an Open Science mindset, including information on how to collaborate on the project and the contact information of the authors is also desirable.

Other aspects of the project, such as a list of contributors, may also be included in the README file. The README file may also be called DESCRIPTION, although README is a much more widely accepted standard.

Additional documentation can be added to the project in several ways (see Figure 1). A common documentation file is the CONTRIBUTING file, which contains information on how to contribute to the project, how authorship of eventual publications will be assigned, and other community-level information. The CODE_OF_CONDUCT file contains guidelines and policies on how the project is managed, the expected conduct of project members, and potentially how arising issues between project members are resolved. Such a file can be important to either projects open to collaboration from the public or large consortium-level projects. Another important documentation file in the Open Source community is the CHANGELOG file. It contains information on how the project changed over time and its salient milestones. For data analysis, it could be used to inform collaborators of important changes in the codebase, methodology, or any other news that might be important to announce and record. Additionally, together with the commit history, CHANGELOG files can be useful for tracking the provenance of the analysis, as we have already mentioned.

A common place to store documentation is the top level of the project repository, but some templates use the docs folder, also from guidelines used in the Python community (to use tools such as Sphinx ^14^).

We can conclude by reiterating that the second principle states that **documentation is essential**.

*3. Be logical, obvious, and predictable*

When a project layout is logical, obvious, and predictable, human users can easily and quickly understand and interact with it.

To be *logical*, a layout should categorize files based on their content and logically arrange them according to such categories. To be *obvious*, this categorization should make sense at a glance, even for non-experts. For instance, a folder named “scripts” should contain scripts (to be obvious) and only scripts (to be logical). To be *predictable*, a layout should adhere to community standards, so that it “looks” similar to other projects. This creates minimal friction when a user first encounters the project and desires to interact with it.

This principle is also present in aspects of project structure other than layout. For instance, the structure of documentation can also benefit from the same principles but in a different context: logically arranged, obvious in structure, and similar to other projects.

This might be the most difficult principle to follow because it largely depends on the community as a whole. For this reason, we hope that the analysis shown above, especially in Figure 1, and our proposed minimal structure (presented in the next sections) will be useful as guides to effectively implement this principle.

An additional benefit of standardizing project structure is that it allows for tools to leverage it, helping data analysts in repetitive tasks. For example, if all log files created during a workflow run were to be saved in the same folder, a tool could be configured to quickly delete them if the need arises. Were such files dispersed among input, output and even source file, this task may be more difficult and/or error-prone.

We can summarize this third principle like this: **be logical, obvious, and predictable**.

*4. Promote (easy) reproducibility*

Scientific Reproducibility has been and still is a central issue, particularly in the field of biomedical research. ^15, 16^ Scientific software developers hold crucial responsibility toward the scientific community of creating reproducible data analysis software.

“Reproducibility” can be understood as the ability of a third-party user to understand the research issue investigated by the project, how it was addressed, and practically execute the analysis proper again to obtain a hopefully similar and ideally identical result to the original author(s). This has two benefits: a reproducible analysis evokes more confidence in those who read and review it, and it makes it much easier to repurpose the analysis to similar data in the future.

In the modern era, scientists are equipped with powerful tools to enable reproducibility, such as containerization and virtualization. While a discussion on how reproducibility can be achieved eludes the scope of this article, the project layout can promote it, especially when all other principles presented here are respected. This increased adoption can be promoted by including obvious and easily implementable reproducibility methods in the project layout directly.

Workflow managers, such as Nextflow, ^6^ Snakemake, ^7^ and the Common Workflow Language (CWL), ^8^ are key tools to enable reproducibility. They allow a researcher to describe in detail the workflow used, from input files to the final output, offloading the burden of execution to the workflow manager. This allows greater transparency in the methodology used and even makes reproducibility a possibility in more complex data analysis scenarios. Additionally, some workflow managers are structured to promote the reusability of the analysis code, even in different architectures or high-performance computing environments. ^8^

Usage of these tools requires specific training. Tools like CWL are invaluable when performing complex, distributed data analysis tasks that require leveraging, for instance, distributed, high-performance computing infrastructures. In these cases, bioinformaticians and other data analysts would immediately see the inherent value of such technologies. However, untrained or low-skill analysts might not be aware of, or able to, use them. As we already mentioned in the Introduction, simple tools with low entry barriers would render reproducibility more accessible.

We conclude this section by stating the fourth and last principle: **be (easily) reproducible**.

### Kerblam!

We designed a very simple but powerful and flexible project layout together with a project management tool aimed at upholding the principles outlined in the previous section. We named this tool “Kerblam!”.

**Figure 2.**
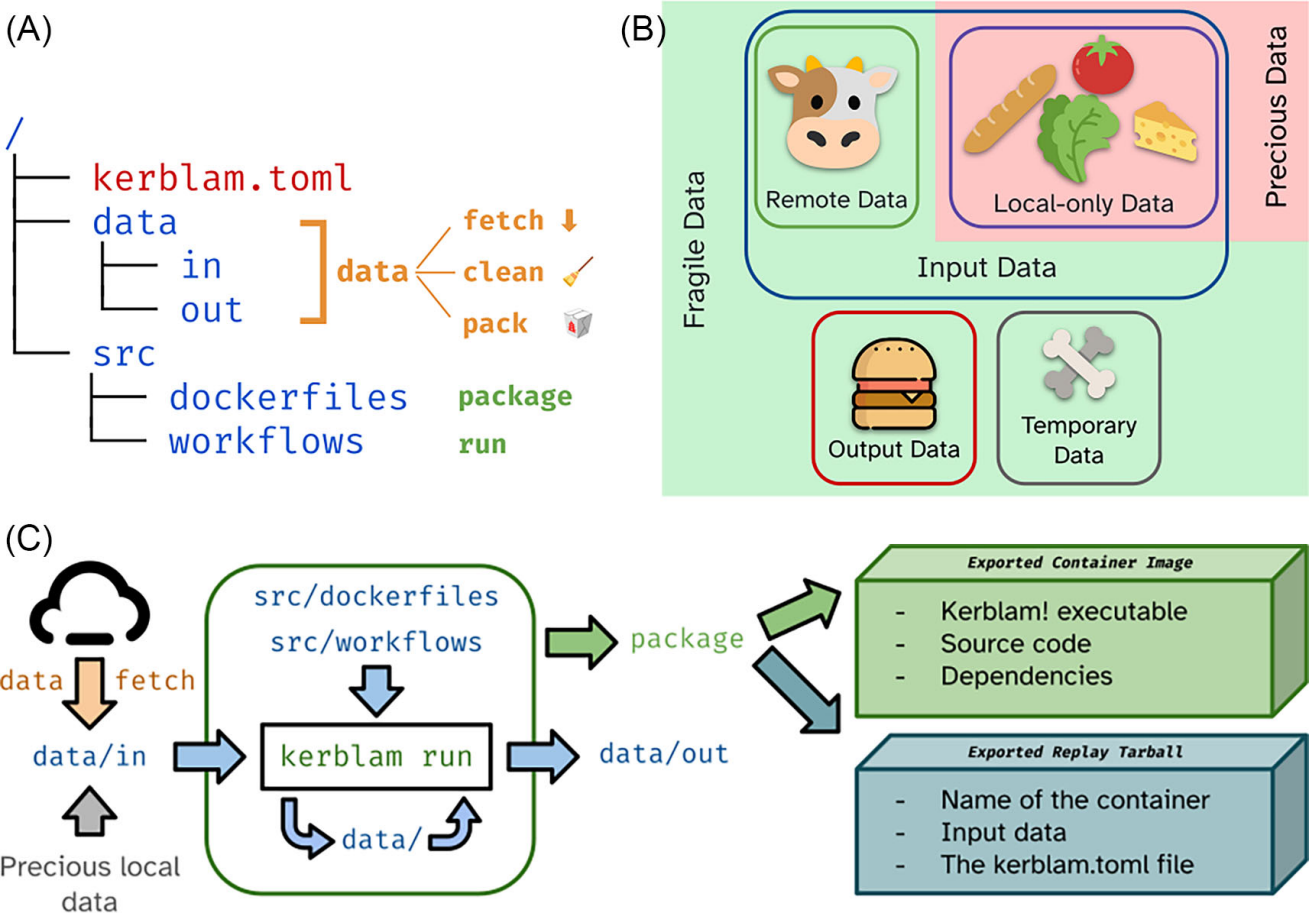
Salient concepts implemented by Kerblam! (A): Basic skeleton of the proposed folder layout for a generic data analysis project associated with relevant Kerblam! commands. Folders are depicted in blue, while files are depicted in red. (B): Data is qualitatively divided into input, output, and temporary data. Input data can be further divided into input data remotely available (i.e., downloadable) and local-only data. The latter is “precious”, as it cannot be easily recreated. Other types of data are “fragile”, as they may be created again on the fly. (C): Overview of a generic Kerblam! workflow.

In particular, Kerblam! encourages the use of version control with Git (principle 1), allows documentation to be written near the code that it describes (principle 2), invites the analyst to use a simple, logical, obvious and predictable folder structure (principle 3) and makes using containerization tools such as Docker easier and less burdensome by the end user (principle 4). Additionally, it encourages the use of remote file storage (pushing for FAIR-er data), and allows researchers to create and publish readily executable container images to the public to re-run pipelines for reproducibility purposes (see Figure 2C).

The most basic skeleton of the project layout implemented by Kerblam! is shown in Figure 2A. The kerblam.toml file contains configuration information for Kerblam! and marks the folder as a Kerblam-managed project. Kerblam! provides a number of utility features *out of the box* on projects that adapt to the layout presented in Figure 2A or any other project structure after proper configuration.

*Data management*

Kerblam! can be used to manage a project’s data. It automatically distinguishes between input, output, and intermediate data, based on which folder the data files are saved in: the data folder contains intermediate data produced during the execution of the workflows, the data/in contains input data, and similarly, data/out contains output data. Furthermore, the user can define in the kerblam.toml configuration which input data files can be fetched remotely and from which endpoint. This allows Kerblam! to both fetch these files upon request ( kerblam fetch) and distinguish between remotely available input files and local-only files. Local-only files are deemed “precious” because they cannot be recreated easily. All other data files are “fragile,” as they can be deleted without repercussion to save disk space ( Figure 2B).

These distinctions between data types enable further functions of Kerblam!. kerblam data shows the number and size of files of all types to quickly check how much disk space is being used by the project. Fragile data can be deleted to save disk space with kerblam data clean and precious input data can be exported easily with kerblam data pack. kerblam data pack can also be used to export output data quickly to be shared with colleagues.

Allowing Kerblam! to manage the project’s data using these tools can offload several chores, usually performed manually by the experimenter.

*Workflow management*

Kerblam! can manage multiple workflows written for any workflow manager. At its core, it can spawn shell subprocesses that then execute a particular workflow manager, potentially one configured by the user. This allows Kerblam! to manage *other* workflow managers, making them transparent to the user and with a single access point.

Kerblam! can also act before and after the workflow manager proper to aid in several tasks. First, it can manage workflows in the src/workflows folder *as if* they were written in the root of the project. This is achieved by moving the workflow files from the said folder to the root of the repository *just before* execution. This allows for slimmer workflows that do not crowd the root of the repository or conflict with each other, thus being more consistent.

Second, it allows the concept of *input data profiles.* Data profiles are best explained using an example. Imagine an input file, input.csv, containing some data to be analyzed. The experimenter may wish to test the workflows that they have written with a similar, but, say, smaller test_input.csv. Kerblam! allows hot-swapping of these files just before the execution of the workflow manager through profiles. By configuring them in the kerblam.toml file, the experimenter can execute a workflow manager (with kerblam run) specifying a profile: Kerblam! will then swap these two files just before and just after the execution of the workflow to seamlessly use exactly the same workflow but with different input data, in this case for testing purposes.

Kerblam! supports *out of the box* GNU make as its workflow manager of choice ( https://www.gnu.org/software/make/). Indeed, makefiles can be run directly through it, with no further configuration by the user. Any other workflow manager can be used by writing tiny shell wrappers with the proper invocation command. The range of workflow managers supported out of the box by Kerblam! could increase in the future.

To run workflows, Kerblam! simply creates subshells, executes the proper commands to launch the workflow (or the user-configured shell script), and eventually pipes the outputs of the subshell to the parent shell. This leaves complete control of the workflow to the workflow manager, making the Kerblam! integration seamless.

*Containerization support*

Containers can be managed directly using Kerblam!. By writing container recipes in src/dockerfiles, Kerblam! can automatically execute workflow managers inside the containers, seamlessly mounting data paths and performing other housekeeping tasks before running the container. As previously stated, Kerblam! works “above” workflow managers. Therefore, the reader might question the usefulness of a containerization wrapper at the level of Kerblam! if the workflow manager of choice already supports it. This containerization feature is meant to be used when a workflow manager would be inappropriate. For instance, very small analyses might not warrant the increased development overhead to use tools such as CWL. Kerblam! allows even shell scripts to be containerized anyway, making even the smallest analyses reproducible.

With these capabilities, Kerblam! promotes reproducibility and allows experienced and inexperienced users alike to perform even the simplest analyses in a reproducible manner.

*Pipeline export*

Workflows managed by Kerblam! with an available container can be automatically exported in a reproducible package through kerblam package. This creates a preconfigured container image ready to be uploaded to a container registry of choice together with a compressed tarball containing information on how to (automatically) replay the input analysis: the “replay package”.

The process automatically strips all unneeded project files, leading to small container images.

The replay package can be inspected manually by a potential examiner and either re-run manually or through the convenience function kerblam replay, which recreates the same original project layout, fetches the input container, and runs the packaged workflow.

*The Kerblam! analysis flow*

Kerblam! favors a very specific methodology when analyzing data, starting with an empty git repository. First, upload the input data to a remote archive (in theory, promoting FAIR-er data). Then, configure Kerblam! to download the input data and write code and workflows for its analysis, potentially in isolated containers or with specific workflow management tools. During development, periodically clean out intermediate and output files to check whether the correct execution of the analysis has become dependent on the local-only state. Finally, package the results and pipelines into the respective environments and share them with the wider public (e.g., as a GitHub release or in an archive such as Zenodo).

We believe that this methodology is simple yet flexible and robust, allowing for high-quality analyses in a wide variety of scenarios. This workflow can also be thought of as “stateless”, as it attempts to steer the user away from storing and relying upon previous runs of the same workflows. This philosophy makes replication easier and less error-prone as, essentially, every workflow run is independent from any other.

This “analysis flow” contrasts with other tools available online that attempt to solve or ameliorate the same issues that Kerblam! addresses. We have selected two of them to highlight these differences: “data science operations”, or DSO ( https://github.com/Boehringer-Ingelheim/dso) and “Data Analysis Project management”, or DAP ( https://github.com/molinerisLab/dap).

DSO, similarly to Kerblam!, leverages other tools to provide its functions, such as Data Version Control (DVC, https://github.com/iterative/dvc), Quarto ( https://quarto.org/), and Git ( https://git-scm.com), among others. It revolves around the concept of “stage”, a single step of the analysis workflow, with predefined inputs and outputs. To isolate each step, it creates individual “stage folders” with input, output, source and report folders as well as configuration files. By leveraging extensive configuration and DVC, the authors of DSO seem to prefer an analysis flow that preserves and version-controls both code and data, syncing them both with remote endpoints for collaboration. DVC, in particular, is aimed at the management of Machine Learning models. In such models, recording exactly which input data was used to train a specific version of a model is essential, so such an approach may be warranted. Indeed, DVC lets users “commit” and remotely store data similarly to code. However, we argue that for most data analysis tasks such versioning is not necessary, as the input data stays largely the same and the computational demands of the analysis are not so high to require careful storage of all output artifacts.

DAP focuses on the Python programming language, leveraging Anaconda ( https://anaconda.org/) for environment management and Snakemake ^7^ for workflow management. DAP also uses direnv ( https://direnv.net/) to update PATH variable with useful shortcuts. DAP creates two main folders to structure the project: a “workflow” folder, with code and configuration files, and a “workspaces” folder, with different project “versions”. DAP “versions” fulfill similar goals as DSO/DVC commits do. Each version of the project is a different workspace folder, which contains symbolic links to a specific (sub) set of files in the “workflow” folder for this specific “version” of the project. A DAP project also uses Git ( https://git-scm.com/) to provide overall version control. Similar to DSO, by laying out different versions of the same project next to each other, DAP also promotes a “state-based” workflow of sorts. When changes to workflows are required, the whole project is copied over, edited, and preserved alongside older copies.

While this is indubitably valuable if one often needs to refer to previous versions of the project, we believe it adds an additional layer of complexity in file and folder structure. This is especially due to the extensive usage of symbolic links, references and other opaque techniques that, while increasing development speed, might hinder the accessibility of the project to new collaborators or reproducers.

### Issues and limitations

Kerblam! is a command-line tool. This means that experience with command line tools is required to use it. This is indeed quite an obstacle when approaching researchers not trained in computer science, as the real and perceived complexity of the command line triggers repudiation of the whole practice. The development of a graphical user interface, as well as interactive and accessible training materials may help surmount this barrier.

Similarly, the usage of Git from the command line may incur the same issues. Fortunately, many graphical interfaces to Git exist. The Git website hosts a list of them, divided by operating system: https://git-scm.com/downloads/guis.

While external dependencies were kept at a minimum, the installation of Kerblam! still requires the user to autonomously install other tools, such as Git and Docker. This might provide additional friction when beginning to use the tool. Methods to completely overcome these issues are unclear.

*Availability*

Kerblam! is a free and open-source software available on GitHub at https://github.com/MrHedmad/kerblam. It is written in Rust and may be compiled to support GNU/Linux-flavored operating systems, MacOS, and Windows. Alternatively, GitHub releases provide precompiled artifacts for both operating systems. The full documentation of Kerblam! is available at https://kerblam.dev. Active support for Kerblam! and its development are guaranteed for the foreseeable future.

## Conclusions

Structuring data analysis projects is a personal matter that is heavily dependent on the preferences of the individuals who conduct the analysis. Nevertheless, best practices arise and can be individuated in this fragmented landscape.

In this study, we aimed to provide such guidelines and include a robust tool to leverage the regularity of such standardized layout. As the proposed layout is, for all intents and purposes, largely arbitrary, Kerblam! can be configured to operate in any layout.

Through these and potentially future standardization efforts, tools such as containerization and workflow managers can become more mainstream and even routine, leading to an overall more mature and scientifically rigorous way to analyze data of any kind.

## Author’s contributions

Conceptualization: L.V., L.M., and F.A.R.; Software: L.V.; Methodology: L.V. and F.A.R; Funding Acquisition: L.M.; Writing - Original Draft Preparation: L.V., L.M., and F.A.R.; Supervision: L.M. and F.A.R.

## Data Availability

The raw data fetched by the analysis of project templates (e.g., list of fetched repositories, detected frequencies) are available on Zenodo. Zenodo: Archival data for Kerblam Project structure. 10.5281/zenodo.13627213. ^17^ This project contains the following underlying data:
•
data_cookies.json. The list of repositories as fetched by the Github Cli utility 2.55.0 on 2024-07-12 with the command gh search repos cookiecutter data --sort stars --json stargazersCount,url --visibility public -L 50
•
data_generic.json. The same as above, with the command gh search repos research project template --sort stars --json stargazersCount,url --visibility public -L 50
•
repos.tar.gz. The resulting (fetched) repositories •
data.json. The combination of data_cookies.json and data_generic.json
•
plot.png and plot.pdf. The plots generated with the information in results.csv
•
results.csv. The result of the folder and file enumeration of the repositories in the repos.tar.gz file, with the following columns:
○
path. The full path from the root of the repos directory to the file ○
count. The frequency of this specific item in the various repositories ○
types. An enumeration of either “directory” for directories or “file” for files Data are available under the terms of the Creative Commons Zero “No rights reserved” data waiver (CC0 1.0 Public domain dedication).

## Software availability statement

The code for the analysis is available on GitHub and is archived on Zenodo.

• Source code available from: https://github.com/MrHedmad/ds_project_structure
• Archived source code available from: https://doi.org/10.5281/zenodo.14611208 Luca “Hedmad” Visentin. (2024). MrHedmad/ds_project_structure: Project Structure version 2 (Version 2). Zenodo.
• License: MIT License.

Kerblam! is available on GitHub and archived at every release in Zenodo.

• Source code available from: https://github.com/MrHedmad/kerblam and https://kerblam.dev/
• Archived source code available from: https://doi.org/10.5281/zenodo.14528820 Visentin, L. (2024). Kerblam! (v1.2.0). Zenodo.License: MIT License.
