# Supplementary material for: Structuring data analysis projects in the Open Science era with Kerblam!
Source: F1000Res. 2025 Apr 4;14:88. Originally published 2025 Jan 15. [Version 2] doi: 10.12688/f1000research.157325.2 (PMC11880754; doi:10.12688/f1000research.157325.2)
Supplement: Peer review history

# Reviewer report: Reviewer response for version 1

**DOI:** 10.5256/f1000research.172753.r365311

**Published:** 2025-03-04

**Authors:** Priya Silverstein

**Affiliations:** 1 Ashland University, Ashland, USA

Thank you for the opportunity to review this article submitted to F1000 on how data analysis projects are structured. I enjoyed reading the article -- it was well written, concise, and mostly understandable to me despite being well outside of my area of expertise.

 To contextualise my review, I am a psychologist and metascientist who has experience sharing analysis code and data for a variety of projects, but not much confidence in the fact that I have been doing this well, which is one of the reasons I was excited to review this paper! I have used Github, but infrequently, and I'll admit I do not use it in my own usual data analysis management workflow. For these reasons, I think I am unable to give a very detailed or helpful review, due to some of the article going "over my head". However, I hope that different perspective might be in some way helpful, as hearing from those outside of our fields sometimes is. I only have small comments for potential improvement or extension, which the authors can take or leave. 

 1. In the introduction, it would be nice to set up a little more why structuring data analysis projects well is so important, and that one of the reasons is low reproducibility rates. You do of course talk about this in your recommendations, but I think it might also be helpful to lead with the low reproducibility rates in different fields. I'm not sure if any papers have looked at *where* results fail to reproduce, but from trying to reproduce many results myself I know that bad data analysis file structure can definitely contribute to this (for example, if it is not clear which are the final files to be used and where and in which order code should be run). 

 2. I expected a little more discussion of *broadly* the different ways to structure data analysis projects for sharing and their relative pros and cons, for example the difference between sharing your data and code in the same folder (i.e. all the data files that one script calls on together), vs. having data and code stored separately. I think it's also interesting to think about how this intersects with skill level -- because I am a relative novice with R, I feel more comfortable sharing all data and code that will be used together in the same folder, as then I can just have a note in the README that tells people to download the whole folder together and then set the working directory to the source file location when running. 

 3. In general I think this article is pitched to a higher skill level than I possess, so the authors may think about whether this is intentional or whether they are hoping to help and change the behaviours of a wide variety of data analysts. It is OK either way, but will inform the direction the article takes. 

 4. Related to this, I wonder whether the authors wish to keep the article very git-focused (which again, is OK if intentional) or whether there could be a brief mention of other types/solutions for version control and their relative barriers of entry.

 5. Lastly, I think the introduction of Kerblam currently feels a little strange -- it is neither the focus of the article that everything else leads up to, nor is it an afterthought, it is kind of in-between. Perhaps the authors could think about how they would like Kerblam to fit into the article and make some adjustments accordingly.

Is the rationale for developing the new method (or application) clearly explained?

Yes

Is the description of the method technically sound?

Yes

Are the conclusions about the method and its performance adequately supported by the findings presented in the article?

Yes

If any results are presented, are all the source data underlying the results available to ensure full reproducibility?

Yes

Are sufficient details provided to allow replication of the method development and its use by others?

Yes

Reviewer Expertise:

Psychology, Metascience

I confirm that I have read this submission and believe that I have an appropriate level of expertise to confirm that it is of an acceptable scientific standard.

# Response

**Published:** 2025-03-25

**Authors:** Luca Visentin

**Affiliations:** Department of Life Sciences, University of Turin, Turin, Italy

We sincerely thank you for your review. We are glad that you found our article relatively accessible, as that was one of our goals when writing it. We are also very happy to receive comments from a researcher such as yourself, as perspectives from non-bioinformaticians on bioinformatics-oriented tools are rare to find.

 We have taken your suggestions into considerations as we drafted a second version of the article. To be more specific, we have expanded the introduction with data from slightly old but popular articles regarding the low rates of reproduction. Although it makes intuitive sense that project structure would play a role in reproducibility rates (or at least that's what we believe), we were unable to find specific numerical data on the contribution of each reproducibility risk factor to the overall reproducibility rate. We cannot therefore reliably conclude how much of an impact project structure standardization would have on reproducibility rates. It would be an interesting future research prospect indeed.

 Related to your second point, our analysis combines different project structures, but does not try to cluster them into "kinds". We believe that this would be a requirement to comment on the pros and cons of different types of project structures. Originally, we considered submitting questionnaires to data analysts and researchers in general regarding the structure they use when dealing with data. This would have been very interesting and would lead to a more detailed and informed discussion about what kinds of project structures are there and how they are used, as well as why researchers use them. However, we determined we did not have the means to reach a large enough sample of researchers from many different backgrounds to do this effectively, so we relied on Github-available data instead although we believe it is an inferior approach.

 Regarding skill level, we have included an explicit reference to it in the fourth principle. We hoped that the article would be interesting to both novices and to expert data analysts, and it was written with this in mind. We believe that your insightful comments are a sign that we managed to do that, but we have nonetheless added an explicit reference to our target audience in the introduction. We have added a section, "Issues and limitations", discussing these barriers of entry.

 The reliance of Kerblam! to Git is indeed by choice: other version control systems exist, but none are as widespread as Git, so we believe that consideration of other VCSs here would be out of scope. In any case, Kerblam! does not *enforce* the usage of Git, it only encourages it. If the users desires to use, for instance, Mercurial as their VCS of choice, they may do so and still use Kerblam! to its full capabilities. In the "Issues and limitations", we mention graphical user interfaces to Git that a user may use instead of the terminal.

 We agree that the introduction to Kerblam! was a bit rough and detached from the rest of the article. We have reworded it significantly in Version 2. We hope it makes the transition a little more fluid.

# Reviewer report: Reviewer response for version 1

**DOI:** 10.5256/f1000research.172753.r363657

**Published:** 2025-02-20

**Authors:** Rodica Cujba

**Affiliations:** 1 Technical University of Moldova, Chișinău, Moldova

The paper focuses on standardizing the structure of data analysis projects to enhance reproducibility and transparency within the framework of Open Science. The study reveals a significant lack of consistency in how researchers organize their data analysis projects. This inconsistency impedes reproducibility and complicates the ability of others to understand and utilize the work. The authors emphasize the importance of aligning data analysis project structures with the FAIR principles (Findable, Accessible, Interoperable, and Reusable). They argue that standardization fosters these principles. The researchers analyzed a large number of data analysis project templates from GitHub to identify common practices and patterns. A frequency analysis of files and folders was performed.

 The paper emphasizes the importance of reproducibility and transparency in Open Science and introduces a practical tool (Kerblam!) to support these goals.

 The study proposes four design principles for creating well-structured data analysis projects: Use a version control system; Documentation is essential; Be logical, obvious, and predictable; Promote (easy) reproducibility. These principles offer a framework for creating more accessible and reusable data analysis projects. The Kerblam! supports these design principles and thus simplifies data management, workflow execution, and containerization.

 The rationale for developing Kerblam! is clearly explained. The paper effectively establishes the need for a standardized approach to data analysis project structuring by highlighting the significant inconsistencies in current practices. The authors explicitly link this lack of standardization to challenges in reproducibility, transparency, and collaboration, which are all fundamental principles of Open Science. They then introduce Kerblam! as a direct response to these issues—a tool designed to promote the four design principles they outlined. The connection between the identified problem and the proposed solution is therefore well-established and convincing.

 The technical description of the Kerblam! method is largely sound, but could benefit from some clarifications and expansions.

 While the paper describes the *functionality* of Kerblam!, more details regarding its *implementation* would strengthen the technical soundness. This could include: 1) Specifics about the Rust implementation (e.g., use of libraries, design patterns); 2) Explanation of how shell subprocesses are managed to interact with different workflow managers;

 Discussion of security aspects (e.g., access control, data encryption) is missing. This would be beneficial for a tool intended for scientific collaborations.

 The paper provides enough information to understand the core concepts and rationale behind Kerblam! and to reproduce the *analysis* of existing project structures.

 The authors state that the raw data underlying their results are available on Zenodo. This is a significant strength, as it allows for full reproducibility of the analysis of existing project templates. The Zenodo link allows a reader to verify the analysis steps and the resulting frequency graph presented in the paper. This commitment to data availability significantly enhances the reproducibility of the research.

 The conclusions are generally supported by the findings, but a comparison with other existing tools or methods for data analysis project management would further solidify the conclusions about Kerblam!'s unique contributions.

Is the rationale for developing the new method (or application) clearly explained?

Yes

Is the description of the method technically sound?

Partly

Are the conclusions about the method and its performance adequately supported by the findings presented in the article?

Partly

If any results are presented, are all the source data underlying the results available to ensure full reproducibility?

Yes

Are sufficient details provided to allow replication of the method development and its use by others?

Yes

Reviewer Expertise:

Open Science, Information Technologies

I confirm that I have read this submission and believe that I have an appropriate level of expertise to confirm that it is of an acceptable scientific standard.

# Response

**Published:** 2025-03-25

**Authors:** Luca Visentin

**Affiliations:** Department of Life Sciences, University of Turin, Turin, Italy

We kindly thank you for your detailed review. We also appreciate your highlighting of the reproducibility of our analysis, and we hope Kerblam! was useful to you in checking it. We have submitted a second version of the article now, hoping to address at least some of your concerns.

 While the article is aimed at researchers somewhat familiar with technical terminology, we wanted to keep it as accessible as possible to a wide range of skill levels. For this reason, we have kept discussions of the implementation details to a minimum in the article. In any case, Kerblam! is open source, and the contributing guide ( https://github.com/MrHedmad/kerblam/blob/main/CONTRIBUTING.md) can be used as an entry point to dive into the implementation. The code is as modular as possible, and we hope that docstrings and various comments make it understandable.

 In the second versions of the paper, we have added a short paragraph specifying how Kerblam! handles starting other workflow managers. We are still, however, not including too many information in this regard due to the aforementioned accessibility concerns.

 Regarding data security and encryption, Kerblam! is an exclusively locally-executed tool that ***is*** specifically used to manage workflows, so encryption is outside of its scope. In any case, cryptographic signing of Kerblam! replay packages is a feature we are currently exploring, so that users can be sure that they are replaying the original replay package.

 It is indeed true that our original paper did not include any comparison with existing tools. In the second version, we have added a new section comparing Kerblam! with similar tools, highlighting its relative strengths and weaknesses.

 We hope you will enjoy the second version of the paper, and we are open to any technical suggestions either here or directly in the Kerblam! repository.
